# The Efficacy of Platelet-Rich Fibrin in the Management of Chronic Nonhealing Ulcers of the Lower Limb

**DOI:** 10.7759/cureus.26829

**Published:** 2022-07-13

**Authors:** Zwalitha Singampalli, Yadavalli R. D. Rajan, Ramavath Hemanth Rathod, Pratti Lohi S RajLaxmi

**Affiliations:** 1 General Surgery, Siddhartha Medical College, Vijayawada, IND; 2 Dermatology, Andhra Medical College, Visakhapatnam, IND; 3 Dermatology, Siddhartha Medical College, Vijayawada, IND

**Keywords:** platelet-rich fibrin, nonhealing ulcers, wound healing, growth factors, prf

## Abstract

Introduction

Nonhealing ulcers have a huge burden on the patient, by having high morbidity in terms of chronic pain, partial or complete loss of function, mental health issues, and social isolation, and can have a massive financial burden on the patient. Various novel therapies have been developed to treat nonhealing ulcers. Platelet-rich fibrin (PRF) has been developed in the recent era initially in the dental world for treating oral ulcers. Now, the role of PRF is strongly established in treating nonhealing ulcers. PRF has an aggregate of a myriad of growth factors and cytokines that stimulate healing of the wounds. In this study, we have compared the efficacy of PRF in treating nonhealing ulcers of the lower limb against normal saline dressings.

Aims and objectives

This study aims to determine the efficacy of platelet-rich fibrin over normal saline dressings in the treatment of nonhealing ulcers of the lower limb by comparing the percentage reduction in the surface area of wounds after treatment in both groups.

Methods

Fifty patients with nonhealing ulcers were selected and randomly divided into two groups with 25 patients in each group. Cases were treated with PRF dressings weekly for a period of six weeks. Controls were treated with normal saline dressings weekly for a period of six weeks. The percentage reduction in the size of the ulcer after treatment was recorded in both groups and compared. Patients of age 18-60 with nonhealing ulcers of the lower limb of >12 weeks duration have been included in the study. Patients having wounds with active infection, wound size > 35 cm^2^, uncontrolled diabetes, peripheral vascular disease with Ankle-Brachial Index < 0.9, osteomyelitis of the underlying bone, and immunocompromised state, and patients on antiplatelet drugs have been excluded from the study.

Results

The mean age of the patients included in the study was 42.88 ± 2.73 years. The mean initial surface of the wound​​​^ ^was 14.95^ ^± 3.08 cm^2 ^among cases and 13.28 ± 2.83 cm^2^ among controls. The mean surface area of the wound after six weeks was 1.59 ± 0.78 cm^2^ among cases and 11.08 ± 2.74 cm^2^ among controls. The mean percentage reduction in the wound size after six weeks of treatment was 89.3% among cases and is significantly higher than in the normal saline group (16.5%) (Mdn = 14.63, U = 23, p < 0.00001).

Conclusion

Platelet-rich fibrin is an emerging potential topical agent for the treatment of nonhealing ulcers of the lower limbs and is more effective than normal saline dressings and also has the advantage of being cost-effective.

## Introduction

Ulcers occur in the lower limb due to a myriad of etiologies, and the treatment of the ulcers depends upon the specific etiology. If the ulcer does not heal within a period of three months or more, they are called nonhealing ulcers [1]. The words “chronic ulcer” and “nonhealing ulcer” have been used interchangeably in this study. Nonhealing ulcers have a huge burden on the patient, by having high morbidity in terms of chronic pain, partial or complete loss of function, mental health issues, and social isolation, and can have a massive financial burden on the patient [2]. Ulcers on the lower limb occur mainly due to trauma, pressure, diabetes, or vascular disorders. Venous ulcers are the most common cause of chronic leg ulcers in India [3]. Platelet-rich fibrin (PRF) is a novel therapy that is being used in the treatment of nonhealing ulcers of the limbs and surgical site wounds. It contains aggregates of a network of platelets and leukocytes that release various growth factors and cytokines, which aid in multiple ways to wound healing [4]. The potential and significance of PRF in treating nonhealing wounds need to be emphasized as there are not many studies regarding the same in this geographical region. In this study, a comparative analysis has been done between platelet-rich fibrin and normal saline dressings in the treatment of nonhealing ulcers of the lower limb to substantiate the efficacy of PRF.

## Materials and methods

This study was conducted at a tertiary care center for a period of two years as a randomized controlled study between autologous platelet-rich fibrin and normal saline dressings in the management of nonhealing ulcers of the lower limb. Patients of age between 18 and 60 and with nonhealing ulcers of the lower limb of >12 weeks duration were included in the study. Patients with wounds with active infection, wound size > 35 cm^2^, uncontrolled diabetes (HbA1c > 9%), peripheral vascular disease with Ankle-Brachial Index < 0.9, and active osteomyelitis of the underlying bone, immunocompromised patients, and patients on antiplatelet drugs were excluded from the study. An institutional review board (Institutional Ethics Committee of Siddhartha Medical College and Government General Hospital (IEC SMC & GGH)) approval was taken (reference number: IEC\2019\033\SMC), and informed consent was taken from all the participants.

Fifty patients with nonhealing ulcers who satisfy the inclusion and exclusion criteria were selected and randomly divided into two groups, group A and group B, with 25 patients in each group. Group A is the study group (PRF group), and group B is the control group (normal saline). The initial surface areas of the wounds were measured at the time of presentation and have been noted before and during the intervention in all the patients. Before the intervention, all the wounds were debrided with sharp surgical debridement. The measurement of the longest dimension is considered as length (l), and the measurement of its perpendicular dimension is considered as breadth (b). Assuming the shape of the wound to be roughly elliptical, the surface area of the wound is calculated using the following formula: length (l) x breadth (b) x (π/4) = surface area of the wound. In group A, after the initial debridement and measuring the surface area of the wound, PRF gel obtained from an autologous blood sample of the patient is spread over the surface of the wound, and a secondary dressing is applied over it. It is left in place for seven days, and the dressing is removed after seven days. This process is repeated for six weeks. The surface area of the wound is measured at the end of every week. In group B, after initial debridement and measuring the surface area of the wound, a moist normal saline dressing is applied over the wound, and the wound is covered with a secondary dry dressing. This dressing is changed every week for six weeks. The surface area of the wound is measured at the end of every week. Data were collected, and the Mann-Whitney U test was used to test the significance (p* *< 0.05) of the percentage reduction in the size of the ulcer in the PRF group compared with the normal saline group. Correlation between the presence of diabetes and hypertension and its effect on the wound healing time was done using the point-biserial correlation test. A linear regression probability test was done to predict a specified time in weeks for the ulcer to heal with PRF therapy depending on the initial surface of the ulcer at presentation.

Method of preparation of PRF

Autologous blood (15 mL) is collected into a sterile test tube without anticoagulant, and the other test tube is filled with 15 mL of normal saline. These tubes are placed in the opposite holes of the metal rotor, and the lid is closed. Centrifugation is done at 3,000 rotations per minute (rpm) for 15 minutes. The tube with the patient’s blood is removed from the machine, and the resultant in the tube contains three layers: a top layer of plasma, a middle layer of PRF gel, and a bottom layer of RBC. The supernatant plasma is discarded, and the PRF gel along with the RBC layer is removed with forceps. The RBC layer is separated from the PRF gel with a sterile blade. The PRF gel thus obtained is applied over the wound.

Method of dressing

The wound is debrided with sharp dissection to remove any necrotic slough or fibrous layer. Thorough saline wash is given to remove any blood clots, and PRF gel is spread over the surface of the wound. A secondary dressing with a sterile gauze pad is placed over this and secured with a roller bandage. This dressing is left intact for seven days and is removed on the eighth day. The patients were asked to take precautions to avoid wetting of the dressing from external sources. At the time of changing the dressing, the wound is checked for any clinical evidence of infection. This process is repeated for a total of six weeks.

## Results

**Figure 1:**
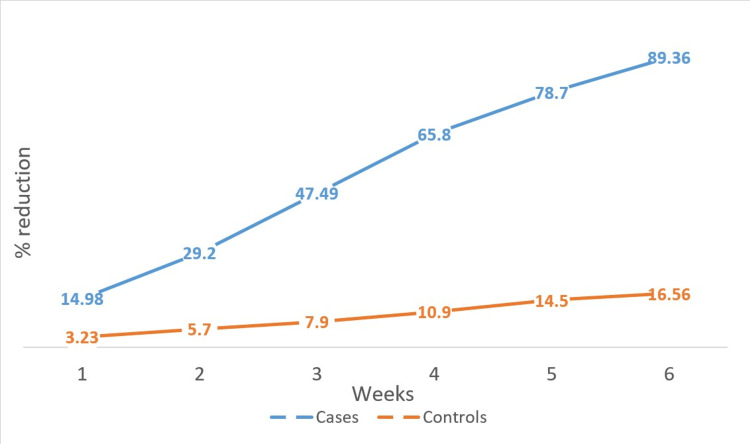
Graphical representation of percentage reduction in the size of the wounds in cases and controls after each week

**Figure 2:**
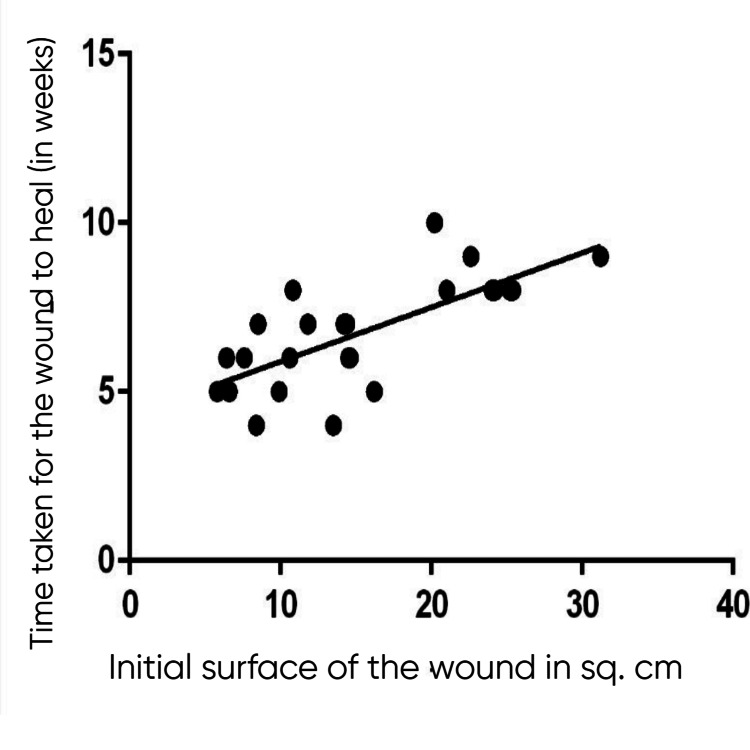
Linear regression probability graph showing the approximate time taken for the wound to heal with PRF therapy in relation to the initial surface area of the wound

**Figure 3:**
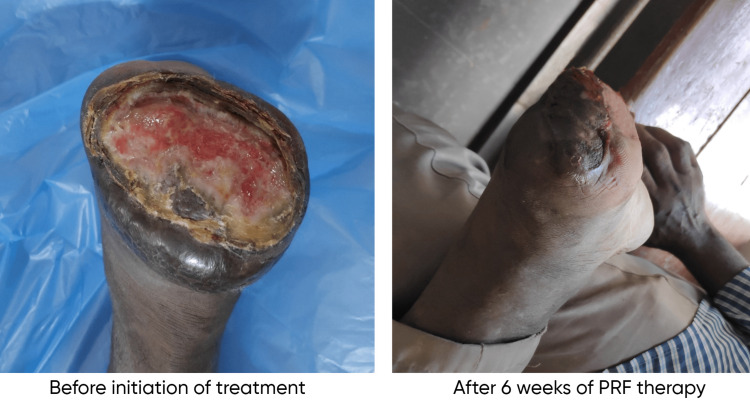
Patient 1 with postoperative (forefoot amputation) nonhealing ulcer over the stump before and after treatment with PRF

**Figure 4:**
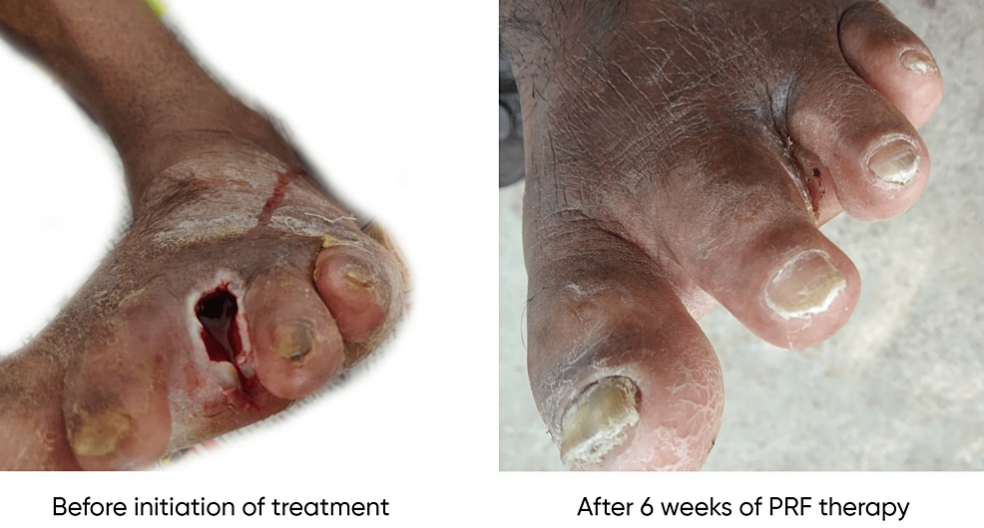
Patient 2 with nonhealing ulcer after ray amputation of the third toe before and after treatment with PRF

The mean age of the patients included in the study was 42.88 ± 2.73 years. Males were 60% (30 patients), and females were 40% (20 patients). The mean initial surface area of the wound was 14.95 ± 3.08 cm^2^ among cases and 13.28 ± 2.83 cm^2^ among controls. The mean surface area of the wound after six weeks was 1.59 ± 0.78 cm^2^ among cases and 11.08 ± 2.745 cm^2^ among controls. The mean percentage reduction in the surface area of the wound after six weeks of treatment was 89.3% among cases and 16.5% among controls (Figure 1). A Mann-Whitney test indicated that the percentage reduction in the size of the ulcer after six weeks of treatment in the PRF group (group A) (Mdn = 92.71) is significantly higher than that in the normal saline group (group B) (Mdn = 14.63) (U = 23, p < 0.00001). The etiologies of the ulcers included in group A were trauma (including one postoperative wound) (20%, 5), venous (40%, 10), diabetic (including one postoperative wound) (24%, 6), and trophic (16%, 4). The etiologies of the ulcers included in group B were trauma (including one postoperative wound) (16%, 4), venous (48%, 12), diabetic (24%, 6), and trophic (12%, 3). The etiology of the ulcers in both groups was almost similar. No signs of infection of the wound were seen in any of the patients. Out of the 25 patients in the study group, 24 ulcers healed completely, and one patient did not turn up for all the treatment sittings (until the ulcer is completely healed). The mean ulcer healing time in the study population among the 24 ulcers that completely healed was 6.58 ± 1.61 weeks. The linear regression probability (Y = 0.16*X + 4.28) between the initial surface area of the wound and the time required to heal in weeks among the case group is shown in Figure 2. The presence of diabetes had no significant effect on wound healing in our population (r = -0.066, p = 0.75). Similarly, the presence of hypertension also did not have any significant effect on wound healing in our population (r = -0.034, p = 0.87). Figure 3 and Figure 4 show the progress of the ulcers treated with PRF in two of the patients.

## Discussion

Wound healing is a complex and dynamic process that includes organized cellular, molecular, and humoral mechanisms. Broughton et al. described it as a complex series of reactions and interactions among cells and mediators [5]. Disruption of any of these factors leads to impaired or delayed wound healing. Briefly, there is initial hemostasis, increased vascular permeability, chemotaxis, and closing of the wound, followed by fibroblast proliferation, re-epithelialization, neovascularization, and ultimately maturation and remodeling of the collagen [6]. Nonhealing ulcers are defined as ulcers that have not responded to initial therapy or have not healed within the given time frame (six weeks in this study) with an underlying etiology such as venous stasis, arterial insufficiency, pressure, and neuropathic ulcers. The general management of nonhealing ulcers includes debridement of the ulcer, inflammation and infection management, moisture control, and environmental and epithelialization assessment. Several newer topical agents such as growth factors, colony-stimulating factors, platelet-derived products, and acellular dermal matrix have been tested for the treatment of nonhealing ulcers.

Due to the virtue of their growth factor and other cytokine content, platelet-derived products are being used in wound healing. Their use is not restricted to a single branch; they are used in surgery, dentistry, oral and maxillofacial surgery, dermatology, and ophthalmology [7]. The cytokines identified in platelets are transforming growth factor-β (TGF-β), platelet-derived growth factor (PDGF), insulin-like growth factor (IGF-I and IGF-II), fibroblast growth factor (FGF), epidermal growth factor, vascular endothelial growth factor (VEGF), and endothelial cell growth factor. These cytokines play important roles in cell proliferation, chemotaxis, cell differentiation, and angiogenesis. The histamine and serotonin released by the platelets increase capillary permeability, which in turn gives inflammatory leukocytes and macrophages improved access to the wound site and results in macrophage activation [8].

Platelet-rich fibrin (PRF) is a second-generation platelet concentrate consisting of a fibrin matrix gel polymerized in a tetra molecular structure, with the incorporation of platelets, leucocytes, cytokines, and circulating stem cells. It is an important advancement in regenerative medicine [9]. PRF was initially developed in the dental world as an inexpensive and user-friendly surgical adjuvant to improve healing and promote tissue regeneration, particularly in oral surgery and implant dentistry [10]. The use of platelet-rich fibrin (PRF) is emphasized for complex wounds as an alternative, simple, inexpensive, time-saving process that does not require hospitalization and has a healing potential over that of bare soft tissue, including bone, tendon, and ligaments [11]. Platelets release the main regulators of angiogenesis, namely, vascular endothelial growth factors, basic fibroblast growth factor (FGF-2), and platelet-derived growth factors, which stimulate angiogenesis. PRF also contains leukocytes, which release various growth factors. The TGF-β, PDGF, VEGF, eotaxin, and CCL-5 released by the leukocytes in the PRF promote local vascularization and tissue repairing [12]. Other growth factors, such as IGF-I, IGF-II, FGF, epidermal growth factor, VEGF, and endothelial cell growth factor, play important roles in cell proliferation, chemotaxis, cell differentiation, and angiogenesis. TGF-β stimulates chemotaxis and mitogenesis on neutrophils, monocytes, and macrophages. VEGF increases vessel permeability and neoangiogenesis. Connective tissue growth factor (CTGF) enhances angiogenetic activity, cartilage regeneration, and fibrosis. Epidermal growth factor (EGF) aids in epithelialization, wound contraction, and remodeling [13]. It also releases anti-inflammatory cytokines IL-4, IL-6, and IL-10, which also have an antimicrobial potential. Platelets are an important source of antibacterial peptides such as fibrinopeptide A and B, thymosin beta-4, platelet basic protein, connective tissue-activating protein-3, and platelet factor-4 [14]. The alpha granules of platelets when activated release molecules with direct microbicidal properties such as reactive oxygen species and kinocidins, thus playing vital roles in the defense against microbes [7]. The fibrin in the PRF and the fibrinogen degradation products enhance neutrophil migration and accentuate chemotaxis by overexpressing the membrane receptors CD11c/CD18 [15]. In PRF, the fibrin formation is physiological and forms a tridimensional mesh that supports the platelets and stem cells ideal for concentration at the site of the wound [12]. In vitro, PRF has a strong effect on cell cultures, with stimulation of the proliferation of all the cell lines that have been tested, including fibroblasts, prekeratinocytes, preadipocytes, osteoblasts, and mesenchymal stem cells, and also stimulates the differentiation of bone cells [11]. All these properties are put together to form an integrated process that ultimately stimulates and fastens the wound healing process.

Ozer et al. conducted a study in Turkey among 17 patients. They used PRF for the treatment of nonhealing wounds. All wounds showed significant improvements after PRF therapy. Full closure was observed after a median of 18 months [11]. In a study conducted in Egypt by Goda et al., a total of 36 patients with venous ulcers were divided into the PRF group (18 patients) and the conventional dressing group (18 patients). At the end of four weeks, there has been a statistically significant difference in the percentage reduction of the ulcer between the two groups with better closure in the PRF group [16]. In an auto-controlled prospective cohort study done by Pinto et al., of the 44 nonhealing ulcers treated with PRF, 29 ulcers (17 venous leg ulcers, 10 diabetic ulcers, and two complex wounds) showed full closure in three months, and the remaining ulcers showed significant improvement in the size of the ulcer [17]. The mean percentage reduction in the surface area of the ulcers at the end of six weeks in our study was similar to the results of the other studies. All these studies showed a significant reduction in the size of the ulcer with PRF application over the wound, thus laying a strong foundation for conducting further large-sized trials. Chen et al. have done a systematic review and meta-analysis with eight randomized controlled trials on the effect of PRF in the treatment of chronic nonhealing wounds compared with the routine treatment for chronic nonhealing wounds. They have described complete epithelialization as the definition for complete healing of the wound. All these articles were published from 2016 to 2020. A total of 578 patients were included, with a mean follow-up of 8.3 weeks. Few studies reported the number of completely healed wounds. Of those, 337 patients with venous, diabetic, and other etiologies reported complete healing of the wounds. Statistical analysis showed that for both venous and diabetic ulcers, the number of ulcers that have completely healed in the platelet-rich fibrin group is significantly higher than in the control group (p < 0.05). Three studies have reported a percentage reduction in the size of the ulcer at the end of the treatment period. A total of 85 patients were included in that study, with varied etiology of nonhealing ulcers. The percentage reduction in the size of the ulcer was significantly higher in the PRF group compared with the control group (p < 0.05). Three studies compromising 352 patients have reported complications of infection during the study. The complication of infection in the PRF group was not higher than the complication of infection in the control group (p > 0.05). This article clearly shows the efficacy of PRF over conventional dressings for the treatment of chronic ulcers. This is similar to the results attained in our study [18]. de Carvalho et al. have done a systemic review of the randomized controlled trials where PRF was used to treat wounds of any size and etiology compared with conventional dressings. They considered 10 trials after the elimination of others. Of the 172 patients, 130 patients showed partial or complete healing of the wound with PRF therapy. Out of the 10 trials, eight trials showed platelet-rich fibrin to be effective. This is attributed to the positive impact of platelet-rich fibrin in the wound healing process [19].

Limitations

Since the sample size of this study is less, large-scaled studies should be conducted for the strong establishment of the role of PRF in wound healing. The site of the ulcer can affect the healing, and it has not been considered in the study.

## Conclusions

A significant reduction in the size of the nonhealing ulcers was observed in those treated with PRF when compared with those treated with regular normal saline dressings. This can be attributed to the multiple growth factors and cytokines released by the PRF that stimulates and fastens the wound healing process. The sample size is small in this study, and hence, multicentric randomized controlled trials with a large sample size should be conducted to substantiate the use of PRF in wound healing. It can be concluded that platelet-rich fibrin can be considered an emerging potential topical agent for the treatment of nonhealing ulcers of the lower limbs as it is not only clinically effective but is also cost-effective, easily available, less time-consuming, and technically easy to prepare and has no adverse effects.
